# Ultra-high-field sodium MRI as biomarker for tumor extent, grade and IDH mutation status in glioma patients

**DOI:** 10.1016/j.nicl.2020.102427

**Published:** 2020-09-12

**Authors:** Sebastian Regnery, Nicolas G.R. Behl, Tanja Platt, Nina Weinfurtner, Paul Windisch, Katerina Deike-Hofmann, Felix Sahm, Martin Bendszus, Jürgen Debus, Mark E. Ladd, Heinz-Peter Schlemmer, Stefan Rieken, Sebastian Adeberg, Daniel Paech

**Affiliations:** a Department of Radiation Oncology, Heidelberg University Hospital, Im Neuenheimer Feld 400, 69120 Heidelberg, Germany; b Clinical Cooperation Unit Radiation Oncology, German Cancer Research Center (DKFZ), Im Neuenheimer Feld 280, 69120 Heidelberg, Germany; c Siemens Healthcare GmbH, Erlangen, Germany; d Division of Medical Physics in Radiology, German Cancer Research Center (DKFZ), Im Neuenheimer Feld 280, 69120 Heidelberg, Germany; e Division of Radiology, German Cancer Research Center (DKFZ), Im Neuenheimer Feld 280, 69120 Heidelberg, Germany; f Department of Neuropathology, University Hospital Heidelberg, Im Neuenheimer Feld 224, 69120 Heidelberg, Germany; g CCU Neuropathology, German Consortium for Translational Cancer Research, German Cancer Research Center (DKFZ), Heidelberg, Germany; h Department of Neuroradiology, Heidelberg University Hospital, Im Neuenheimer Feld 400, 69120 Heidelberg, Germany; i Faculty of Physics and Astronomy and Faculty of Medicine, University of Heidelberg, Heidelberg, Germany

**Keywords:** AUC, area under the curve, CRT, chemoradiotherapy, GBM, glioblastoma WHO IV, Gdce, Gadolinium-contrast-enhancing regions, IDH, isocitrate dehydrogenase, IQR, interquartile range, LGG, lower-grade-glioma WHO I-III, MGMT, O^6^-methylguanine DNA methyltransferase, ^23^Na, sodium, Nawm, normal-appearing white matter, ROC, receiver operating characteristic, SD, standard deviation, SNR, signal-to-noise ratio, T, Tesla, 3D-DLCS, 3D Dictionary Learning Compressed Sensing algorithm, Glioma, Glioblastoma, IDH mutation, Ultra-high-field MRI, Sodium imaging, Non-invasive biomarker

## Abstract

**Purpose:**

This prospective clinical trial investigated sodium (^23^Na) MRI at 7 Tesla (T) field strength as biomarker for tumor extent, isocitrate dehydrogenase (IDH) mutation and O6-methylguanine DNA methyltransferase (MGMT) promotor methylation in glioma patients.

**Methods:**

28 glioma patients underwent ^23^Na MRI on a 7T scanner (Siemens Healthcare, Erlangen, Germany) parallel to standard 3T MRI before chemoradiation. Areas of Gadolinium-contrast enhancement (gdce), non-enhancing T2-hyperintensity (regarded as edema), necrosis, and normal-appearing white matter (nawm) were segmented on 3T MRI imaging and were co-registered with the ^23^Na images. The median total ^23^Na concentrations of all areas were compared by pairwise t-tests. Furthermore, areas of gdce and edema were merged to yield the whole tumor area without necrosis. Subsequently, the difference in median of the ^23^Na concentration of this whole tumor area was compared between IDH-mutated and IDH wild-type gliomas as well as MGMT methylated and MGMT not-methylated glioblastomas using Whitney-Mann U-tests. All p-values were corrected after the Bonferroni-Holm procedure.

**Results:**

The ^23^Na concentration increased successively from nawm to necrotic areas (mean ± sd: nawm = 37.84 ± 5.87 mM, edema = 54.69 ± 10.64 mM, gdce = 61.72 ± 12.95 mM, necrosis = 81.88 ± 17.53 mM) and the concentrations differed statistically significantly between all regarded areas (adjusted p-values for all pairwise comparisons < 0.05). Furthermore, IDH-mutated gliomas showed significantly higher ^23^Na concentrations than IDH wild-type gliomas (median [interquartile range]: IDH wild-type = 52.37 mM [45.98 – 58.56 mM], IDH mutated = 65.02 mM [58.87–67.05 mM], p = 0.039). Among the glioblastomas, there was a trend towards increased ^23^Na concentration in MGMT methylated tumors that did not reach statistical significance (median [interquartile range]: MGMT methylated = 57.59 mM [50.70 – 59.17 mM], MGMT not methylated = 48.78 mM [45.88 – 53.91 mM], p = 1.0).

**Conclusions:**

^23^Na MRI correlates with the IDH mutation status and could therefore enhance image guidance towards biopsy sites as wells as image-guided surgery and radiotherapy. Furthermore, the successive decrease of ^23^Na concentration from central necrosis to normal-appearing white matter suggests a correlation with tumor infiltration.

## Highlights

• MRI derived total ^23^Na concentration differs significantly in glioma subregions.
• Total ^23^Na concentration could reflect IDH mutation status and tumor grade.
• ^23^Na MRI yields potential non-invasive biomarkers for the treatment of gliomas.

## 1 Introduction

Gliomas represent the most frequent primary malignant brain tumors among adults (Ostrom et al., 2017, Louis et al., 2016). Unfortunately, tumor control using multimodal therapeutic strategies is impeded by the diffuse infiltration of gliomas beyond contrast-enhancing regions on clinical MRI already at an early stage of disease (Niyazi et al., 2016, Eidel et al., 2017, Earnest et al., 1988). In general, prognosis of gliomas is unfavorable but differs greatly (Ostrom et al., 2017). The variety of individual patient prognosis strongly depends on tumor grade (Ohgaki and Kleihues, 2005) as well as genetic features such as isocitrate dehydrogenase (IDH) mutations or O^6^-methylguanine DNA methyltransferase (MGMT) promotor methylation (Olar et al., 2015, Hegi et al., 2005). While IDH mutation is currently regarded as the predominant prognostic factor(Olar et al., 2015), MGMT promotor status predicts the efficacy of chemotherapy and may therefore already be used to select different therapy strategies (Wick et al., 2012, Perry et al., 2017). Hence, the development of imaging methods that yield non-invasive biomarkers of tumor infiltration as well as predictors of histopathological features are highly desirable to support image-guided biopsies and image-guided therapy strategies as well as patient follow-up where serial biopsies are not feasible. During the last decade, advances of ultra-high-field (UHF) MRI techniques have been made that offer increased signal-to-noise ratios (SNR) in X-nuclei MRI (Shah et al., 2016). A promising technique in this field is sodium (^23^Na) imaging which has shown to correlate with the IDH mutation status in glioma patients (Biller et al., 2016, Shymanskaya et al., 2019) and has been used to monitor changes in brain tumors during radiotherapy (RT) (Huang et al., 2018, Thulborn et al., 2019). The purpose of this study was to investigate ^23^Na MRI at 7 Tesla (T) as a novel diagnostic tool in a prospective cohort of 28 glioma patients prior to chemoradiotherapy (CRT). We hypothesized that the quantitative ^23^Na signal correlates with tissue-specific tumor compartments and serves as a non-invasive predictor of tumor grade, IDH mutation and MGMT status.

## 2 Patients & methods

### 2.1 Patients

According to the declaration of Helsinki, this study has received approval by the local ethics committee. MRI examinations were performed after written informed consent was obtained from all patients. A total of 30 glioma patients underwent ^23^Na imaging on a 7 Tesla MRI system before CRT parallel to clinical 3T MRI and CT scans for RT planning. Inclusion criteria were age > 18 years, no ferromagnetic or active implants which are not suitable for 7T MRI, histologically proven diffuse glioma, planned (C)RT, residual tumor burden detectable on clinical MRI after biopsy or resection was performed. Two patients were excluded, one due to the diagnosis of pseudoprogression without necessity for RT after tumor board review of all available clinical data and another patient because of a displacement of the ^23^Na reference tubes during imaging. 14 patients presented with recurrent tumors diagnosed histopathologically following re-resection (n = 4) or radiographically according to the updated response assessment in neurooncology (RANO) criteria (n = 10). Patient characteristics are summarized in Table 1.

**Table 1:** **Overview of patient characteristics (N = 28).** (IQR = interquartile range, N = total number, wt = wild-type, MPR = maximum possible resection).

**Age**	**Median [Years]**	**IQR [Years]**
Median Age	53.5	20.3
**Sex**	**N**	**[%]**
Male	16	57.1
Female	12	42.9
**Pathology**	**N**	**[%]**
Oligodendroglioma	4	14.3
Astrocytoma	3	10.7
Glioblastoma	21	75.0
**Grade**	**N | IDH mutated | IDH wt**	**[%]**
I	1| 0 | 0	3.6
II	3 | 3 | 0	10.7
III	3 | 2 | 0	10.7
IV	21 | 1 | 18	75%
**MGMT-Methylation**	**N**	**[%]**
methylated	14	50
not methylated	4	14.3
not available	10	35.7
**Relapse**	**N**	**[%]**
Newly diagnosed	14	50.0
Relapse	14	50.0
**Surgery**	**N**	**[%]**
Biopsy only	6	21.4
MPR	22	78.6

### 2.2 Sodium MRI

^23^Na imaging was performed on a 7T research scanner (Siemens Healthcare, Erlangen, Germany) using a double-resonant (^1^H/^23^Na) quadrature birdcage coil (RAPID Biomedical, Rimpar, Germany). ^23^Na data was acquired with a density-adapted 3D radial pulse sequence (Nagel et al., 2009) with a nominal spatial resolution of (3 mm)^3^ (N_projections_ = 4000; TR/TE = 160 ms / 0.35 ms, T_acq_ = 10:40 min, T_readout_ = 10 ms). TE was measured as the time difference between the start of the readout and the center of the rectangular 600 µs RF pulse. Image reconstruction was performed with an iterative 3D Dictionary Learning Compressed Sensing algorithm (3D-DLCS) (Behl et al., 2016) (block size B = 3 × 3 × 3; dictionary size D = 80; sample number N_samp_ = 500,000; regularization weighting factor µ = 0.5).

Total ^23^Na concentration was obtained using two reference vials (0.3% and 0.6% NaCl). B_1_^+^ and B_1_^−^ corrections were applied to cope with transmit and receive inhomogeneities. In our case we used the double angle method for the field estimations (Insko and Bolinger, 1993). Because a birdcage coil was used, the principle of reciprocity can be applied (B_1_^+^ = B_1_^−^) (Hoult, 2000).

### 2.3 Clinical 3T MRI and histopathological parameters

All patients received standard of care MRI at a field strength of 3T. This included pre- and post-contrast T1-weighted imaging employing Gadolinium-based contrast agents as well as T2-weighted fluid-attenuated inversion recovery (FLAIR) imaging. Representative MRI protocols are given in supplementary table 1.

Histopathological analysis was performed as part of the clinical routine and encompassed IDH mutation status, as well as MGMT methylation status in the glioblastoma subgroup.

### 2.4 Post-processing

The clinical 3T MR images were co-registered to the RT planning CT scan employing an automatic multi-modal rigid algorithm in MITK (Nolden et al., 2013). Consecutively, an experienced radiologist (D.P., 7 years of experience in neuroimaging) segmented Gadolinium-contrast-enhancing regions (gdce), T2 FLAIR hyperintense non-enhancing regions and necrosis as visible on clinical 3T MRI. Necrosis encompassed all areas of fluid-isointense signal adjacent to gdce (usually located centrally) on visual inspection of T1-weighted post-contrast images. If resection had been performed previously, the corresponding areas were not treated as necrosis but as resection cavities and were not included in the statistical analysis. The T2 FLAIR hyperintense non-enhancing regions were treated as the clinical peritumoral edema. Furthermore, three adjacent image slices with tumor areas were chosen and one representative ROI was placed in contralateral normal-appearing white matter (nawm) on each slice. These ROIs were merged to yield a more robust representation of nawm. Finally, the segmentations and ROIs defined on the clinical MRI were co-registered to the 7T ^23^Na MR-images to extract the ^23^Na concentration values as well as segmentation volumes.

### 2.5 Qualitative assessment

To assess potential differences of the depiction of tumors between clinical 3T FLAIR MRI and 7T ^23^Na imaging, we performed a semiquantitative analysis of the ^23^Na concentration inside the peritumoral edema region. For this purpose, ^23^Na image window was set 2 standard deviations (SD) around the individual mean ^23^Na concentration in the edema (edema mean ± 2 SD) for every patient. Hotspot regions were defined inside the edema segmentation according to two criteria: 1) ^23^Na concentration lies above the upper frame of the window as cut-off value (quantitative) and 2) region is not adjacent to or considerably exceeds the border to the gdce region (qualitative).

### 2.6 Statistics

The median ^23^Na concentration was calculated for each segmentation and ROI. Two-sided, paired t-tests were performed pairwise between all segmentations and ROIs to search for significant differences in the median ^23^Na concentrations between different tissue types. Subsequently, gdce and peritumoral edema were merged resulting in the whole tumor volume segmentation without necrosis or resection cavities. This fusion should include all areas of macroscopically visible tumor infiltration but necrosis to prevent a confounding effect of the high content of cerebrospinal fluid. Whitney-Mann U-tests were used to check a possible difference in the median ^23^Na concentration of the whole tumor volume without necrotic areas between glioblastomas WHO IV (GBM) and non-GBM WHO I-III, IDH-mutated and IDH wild-type (wt) gliomas as well as MGMT methylated GBM versus not-methylated GBM. Additionally, receiver operating characteristic (ROC) curves were plotted to show the prediction of tumor grade, IDH mutation and MGMT methylation by the median ^23^Na concentration inside the whole tumor volume. The subsequent ROC analysis encompassed calculation of the area under the curve (AUC) as well as the best thresholds according to Youden’s index with corresponding sensitivity and specificity values. Moreover, ^23^Na concentration was compared between recurrent and newly diagnosed tumors using a two-sided, unpaired *t*-test. The global level of significance was set to 0.05, and all p-values were corrected according to the Bonferroni-Holm procedure. In addition, further descriptive analyses of the employed imaging techniques were conducted. Firstly, the signal intensities of the different tumor subcompartments on clinical T1- and T2-weighted imaging were normalized to the nawm signal and compared to each other by pairwise t-tests. Secondly, the correlation between segmentation volumes and median total ^23^Na concentrations was investigated using scatterplots and Pearson’s r for each subcompartment (intra-group analysis). Finally, segmentation volumes were compared between different tumor subcompartments by pairwise t-tests and the whole tumor volumes excluding necrosis were compared between histopathological subgroups based on Whitney-Mann U-tests (inter-group analysis). All statistical evaluation employed R version 3.6.0 and the pROC as well as the precrec package (Robin et al., 2011, Saito and Rehmsmeier, 2017).

## 3 Results

**Fig. 1:**
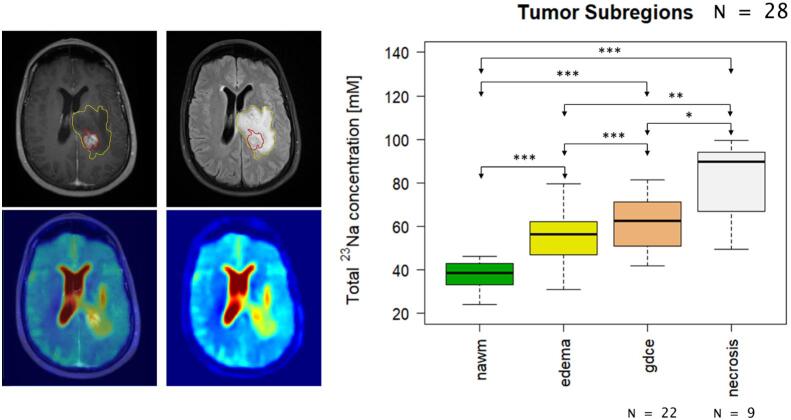
**Total ^23^Na concentration inside different tumor areas. Top left:** T1-weighted imaging after application of Gadolinium-based contrast agent. **Top middle:** T2-weighted FLAIR imaging. **Bottom left:** Fusion of T1-weighted and ^23^Na MRI. **Bottom middle:**^23^Na MRI. The different segmentations are shown on the clinical images: red = Gadolinium-contrast enhancement (gdce), yellow = peritumoral edema. In general, the signal inside the contrast-enhancing region is elevated compared to the non-enhancing peritumoral edema zone. However, there is another hotspot in the peripheral zone of the non-enhancing peritumoral edema, which might reflect an area of increased tumor infiltration. **Right:** the boxplots show the successive increase of ^23^Na signal from normal-appearing white matter (nawm) towards central necrosis (*** = p < 0.001, ** = p < 0.01, * = p < 0.05). (For interpretation of the references to colour in this figure legend, the reader is referred to the web version of this article.)

The comparison of the ^23^Na concentration inside the different tumor subregions and normal-appearing white matter (nawm) showed a successive increase from nawm towards central necrosis (^23^Na concentration mean ± SD: nawm = 37.84 ± 5.87 mM, edema = 54.69 ± 10.64 mM, gdce = 61.72 ± 12.95 mM, necrosis = 81.88 ± 17.53 mM). The concentration differences between all investigated tissue types were statistically significant (see Table 2 and Fig. 1). Moreover, 5 patients presented ^23^Na concentration hotspots inside the peritumoral edema that did not fully correlate with findings on the corresponding clinical FLAIR MRI on qualitative analysis (supplementary table 2 and supplementary Fig. 1).

**Table 2:** **Statistical Results:** All p-values are given as raw values and after Holm-Bonferroni correction to a global α ≤ 0.05 (in brackets). The best cut-offs in the ROC analysis were based on Youden’s index. (N = total number, SD = standard deviation, nawm = normal-appearing white matter, gdce = Gadolinium-contrast enhancement, IQR = interquartile range, AUC = area under the curve, CI = confidence interval, GBM = glioblastoma, IDH = isocitrate dehydrogenase, wt = wild-type, mut = mutation, MGMT = O^6^-methylguanine DNA methyltransferase, met = methylated, ND = newly-diagnosed, MPR = maximum possible resection).

**^23^Na-concentration [mM] in different tumor areas**					
**Region**	**Mean ± SD**	**p-value for pairwise comparison t-Test**			
		**Nawm**	**Edema**	**Gdce**	**Necrosis**
**Nawm (N = 28)**	37.84 ± 5.87	–	5.2 ∙10^−10^**(5.7∙10**^−^**^9^)**	2.7 ∙10^−9^**(2.7∙10**^−^**^8^)**	4.6 ∙10^−5^**(3.7∙10**^−^**^4^)**
**Edema (N = 28)**	54.69 ± 10.64	–	–	6.7 ∙10^−6^**(6.0∙10**^−^**^5^)**	4.3 ∙10^−4^**(0.003)**
**Gdce (N = 22)**	61.72 ± 12.95	–	–	–	0.0078 **(0.039)**
**Necrosis (N = 9)**	81.88 ± 17.53	–	–	–	–

**^23^Na-concentration [mM] and histopathological parameters**					

	**Median [IQR]**	**p-value**	**AUC (95% CI)**	**Best cut-off**	**Sensitivity / Specificity (95% CI)**

**GBM (N = 21)**	54.18 [46.86–58.77]	0.0012 **(0.0074)**	0.89 (0.75–1)	60.39	0.86 (0.57–1.00)/0.90 (0.52–1.00)
**Non-GBM (N = 7)**	66.73 [62.39–67.66]				
**IDH mut (N = 6)**	65.02 [58.87–67.05]	0.0094 **(0.0391)**	0.85 (0.67–1)	55.78	1.00 (0.50–1.00)/0.83 (0.50–1.00)
**IDH wt (N = 18)**	52.37 [45.98 – 58.56]				
**MGMT met (N = 10)**	57.59 [50.70 – 59.17]	0.5395 (1.0)	0.63 (0.23–1)	52.37	0.75 (0.25–1.00)/0.80 (0.10 – 1.00)
**MGMT not-met (N = 4)**	48.78 [45.88 – 53.91]				

**^23^Na-concentration [mM] in recurrent disease**					

	**Mean ± SD**	**p-value**			
**Recurrence (N = 14)**	54.56 ± 10.9	0.5022 (1.0)			
**ND (N = 14)**	57.29 ± 10.4				

**^23^Na-concentration [mM] and different surgical approaches**					

	**Median [IQR]**	**p-value**			
**Biopsy only (N = 6)**	58.35 [57.82 – 59.17]	0.8916 (1.0)			
**MPR (N = 22)**	56.98 [47.55 – 63.18]

Evaluation of the ^23^Na concentration regarding histopathological features revealed a significantly elevated concentration in non-GBM (WHO I-III) compared to GBM (WHO IV) (^23^Na concentration median [IQR]: GBM = 54.18 mM [46.86 – 58.77 mM], non-GBM = 66.73 mM [62.39–67.66 mM], p = 0.0074). Correspondingly, IDH mutant gliomas showed significantly increased ^23^Na concentration as compared to IDH wild-type tumors (^23^Na concentration median [IQR]: IDH wild type = 52.37 mM [45.98 – 58.56 mM], IDH mutated = 65.02 mM [58.87–67.05 mM], p = 0.0391). Hence, when performing ROC analysis, ^23^Na MRI was able to predict tumor grade (AUC = 0.89 [95% CI: 0.75 – 1], sensitivity 85.7%, specificity 90.5%) and IDH mutation (AUC = 0.85 [95% CI: 0.67 – 1], sensitivity 100%, specificity 78%).

**Fig. 2:**
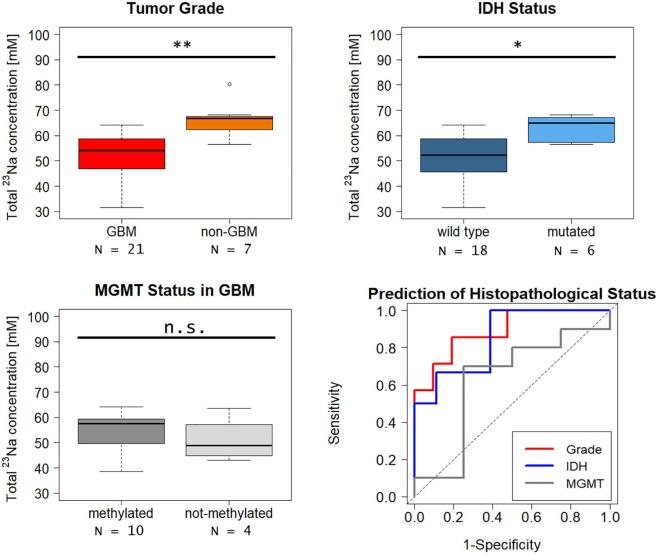
**Total ^23^Na concentration for different grades and IDH mutation status.** Comparison of ^23^Na concentrations for different histopathological grade (**top left**), IDH-status (**top right**) and MGMT-status (**bottom left**) with the corresponding ROC curves (**bottom right**). ^23^Na concentration differs significantly between glioblastoma (GBM, WHO IV) and non-GBM (WHO I-III) with higher values inside non-GBM. Accordingly, significantly higher concentrations can be observed in IDH mutated gliomas. MGMT methylated GBM show a trend towards higher ^23^Na concentration which does not reach statistical significance. The corresponding ROC curves show both high and statistically significant AUC values for prediction of tumor grade (AUC = 0.89) and IDH mutation (AUC = 0.85), but not for MGMT status (AUC = 0.63). (** = p < 0.01, * = p < 0.05, n.s. = not statistically significant).

In the GBM subgroup, MGMT methylated tumors showed a non-significant trend towards increased ^23^Na concentrations (^23^Na concentration median [IQR]: MGMT methylated = 57.59 mM [50.70 – 59.17 mM], MGMT not methylated = 48.78 mM [45.88 – 53.91 mM], p = 1.0) and ^23^Na MRI did not yield a statistically significant predictor of MGMT status in the consecutive ROC analysis (AUC = 0.63 [95% CI: 0.23 – 1]). Fig. 2 yields an overview of the prediction of different histopathological parameters by ^23^Na concentration. Supplementary Fig. 2 shows corresponding precision recall curves.

Newly diagnosed and recurrent tumors did not differ significantly in their ^23^Na signal (^23^Na signal mean ± sd: Recurrent disease = 54.56 ± 10.9 mM, newly diagnosed = 57.29 ± 10.4 mM, p = 1.0). Table 2 summarizes all results of the main statistical analyses.

Supplementary figure 3 and supplementary table 3 give an overview of the normalized signal intensities in different tumor subcompartments on clinical MRI. In contrast to ^23^Na imaging, T1- and T2-weighted sequences could not show a continuous, statistically significant signal change from the center to the peripheral parts of the tumor.

In the additional volumetric analysis, we could not find significant correlations between the volumes and median total ^23^Na concentrations inside different tumor subcompartments (Supplementary Figure 4). The volumes of all subcompartments differed significantly from each other, with peritumoral edema > gdce > necrosis. Furthermore, non-GBM and IDH mutated gliomas showed significantly decreased tumor volumes (Supplementary Figure 5).

## 4 Discussion

In this hypothesis-generating trial, we showed that that the quantitative total ^23^Na signal correlates with tissue-specific tumor compartments and serves as a non-invasive predictor of tumor grade and IDH mutation. No statistically significant concentration differences were found with respect to MGMT promotor methylation.

### 4.1 Origins of the elevated ^23^Na signal

An elevation of the total ^23^Na signal inside gliomas is generally well known (Turski et al., 1987, Hashimoto et al., 1991, Ouwerkerk et al., 2003) and could be explained by the increased intracellular ^23^Na content due to malignant growth (Cameron et al., 1980, Zhu et al., 2016, Rotin et al., 1989) as well as the elevated extracellular volumes in gliomas (Zamecnik et al., 2004, Bruehlmeier et al., 2003, Bakay, 1970). Since the intracellular contribution to the total ^23^Na concentration is relatively small, some authors argue that the elevated ^23^Na signal predominantly reflects an increase in extracellular volume (Thulborn, 2016), whereas others consider both intra- and extracellular changes equally important (Ouwerkerk et al., 2003). The absolute ^23^Na concentrations measured in this study are in good agreement with recent studies at 3T and 4T (Shymanskaya et al., 2019; Thulborn et al., 2019), but almost twofold lower than in an earlier study at 1.5T (Ouwerkerk et al., 2003).

### 4.2 Tumor extent

The observed gradual increase of ^23^Na concentration from nawm towards the central necrotic subregion of the tumor is supported by previous works which found stronger elevation of ^23^Na concentration in gdce as compared to edema without reaching statistical significance (Ouwerkerk et al., 2003, Haneder et al., 2015). This might reflect a tumor infiltration reaching from the border of central necrosis towards the nawm, as previously suggested (Thulborn et al., 2019). Consequently, ^23^Na MRI might yield a quantitative measure of tumor infiltration and thus add more sophisticated information to clinical MRI in treatment planning. However, different biological explanations for elevated ^23^Na concentration than tumor cell infiltration need to be considered: Firstly, non-infiltrative brain edema also leads to higher total ^23^Na levels (Hashimoto et al., 1991, Turski et al., 1986), which might interfere with increased ^23^Na concentration in infiltrated T2 FLAIR hyperintense regions. Similarly, a disruption of the blood brain barrier with consecutively larger extracellular volumes causes elevated ^23^Na concentrations (Turski et al., 1986), which is an alternative explanation to the high ^23^Na levels especially inside contrast-enhancing regions. Ultimately, previous histopathological correlations have suggested that contrast-enhancing regions contain significantly more necrotic areas than non-enhancing regions (Eidel et al., 2017), thus higher ^23^Na concentration inside enhancing regions could also reflect increased amounts of extracellular fluid collections. From a technical perspective, we found that the volumes of all subcompartments differed significantly from each other, which might have affected the measured ^23^Na concentrations. Especially small segmentation volumes could potentially yield a biased estimate of ^23^Na concentration due to partial volume effects (Niesporek et al., 2015, Stobbe and Beaulieu, 2018). However, no significant intra-group correlations were found between the segmentation volume and ^23^Na signal for any subcompartment. Finally, we found several patients with hotspots of ^23^Na concentration inside the tumor edema that did not fully correlate with findings on clinical FLAIR MRI on qualitative assessment. The significance of those hotspots remains unclear, but they could represent zones of increased tumor infiltration. Since a possible correlation of ultra-high-field ^23^Na MRI and tumor infiltration could strongly support image-based treatment planning, this hypothesis should be investigated in forthcoming prospective studies.

### 4.3 Tumor Grade, IDH mutation, and MGMT status

Our findings of significantly increased ^23^Na concentrations in non-GBM (WHO I-III) and correspondingly IDH-mutated gliomas are supported by earlier studies. Those studies investigated the ratio between total ^23^Na signal and intracellular ^23^Na contributions and found a low intracellular to total ratio as predictor of IDH mutation and even progression free survival (Biller et al., 2016, Nagel et al., 2011). The low intracellular to total ^23^Na signal ratio is in line with the elevated total ^23^Na signal found in our study. Furthermore, a recent study at 4T in a smaller patient cohort (n = 11) (Shymanskaya et al., 2019) found significantly increased ^23^Na levels inside IDH-mutated gliomas. The higher ^23^Na concentration in lower-grade gliomas is somewhat counter-intuitive from a biological standpoint because earlier studies suggested a positive correlation between proliferation rate and intracellular ^23^Na levels in various tumor cell lines (Cameron et al., 1980, Zhu et al., 2016, Nagy et al., 1983). However, the intracellular ^23^Na concentration might play a minor role in the constitution of the total ^23^Na signal (Thulborn, 2016). One possible explanation for the elevated ^23^Na signal in non-GBM could be that not only the volume, but also the matrix of the extracellular spaces differs between low- and high-grade gliomas (Zamecnik et al., 2004). This could also influence ^23^Na as the most important counter ion. Moreover, we found that non-GBM and correspondingly IDH mutated gliomas showed significantly decreased tumor volumes. As was already discussed for the tumor subcompartments, a volumetric difference might have affected the measured ^23^Na concentration.

We could not find a statistically significant difference of ^23^Na concentration between MGTM-methylated and MGMT-non-methylated GBM, but the MGMT methylated tumors trended towards increased ^23^Na values. Even though this finding was based on the analysis of a small subgroup (n = 14), it might further support the hypothesis that higher total ^23^Na concentrations reflect a more favorable tumor biology and, therefore, merits further investigation in larger patient cohorts.

### 4.4 Limitations

Our study has several limitations that need to be acknowledged.

1. The heterogeneous patient cohort consisting of recurrent and newly diagnosed tumors is a limitation. However, we additionally compared recurrent to newly diagnosed tumors which did not yield a significant difference of ^23^Na signal among the two groups. This renders an influence of tumor relapse on the ^23^Na concentrations unlikely.
2. Regarding the relatively small and imbalanced patient cohort, the predictive value of ^23^Na MRI for tumor grade and IDH mutation found in this study needs validation in future trials with higher patient numbers. Additional precision-recall analysis hinted towards a somewhat decreased precision due to imbalanced groups, but still suggested ^23^Na concentration as good-fair predictor of tumor grade and IDH mutation.
3. Partial volume effects originating from both, the large voxel sizes and the broad shapes of the point spread function of ^23^Na MRI, were not corrected in this study. Concentration values, especially for small regions, might be affected by surrounding tissues (Niesporek et al., 2015, Stobbe and Beaulieu, 2018).
4. Finally, 7-Tesla MRI scanners, which are especially advantageous for X-nuclei imaging, are not part of the clinical routine in most hospitals. Yet, their availability is steadily increasing since vendors recently released scanners for clinical use, including ^23^Na imaging.

## 5 Conclusion

^23^Na MRI correlates with the IDH mutation status and could therefore enhance image guidance towards biopsy sites as well as image-guided surgery and radiotherapy. Furthermore, the successive decrease of ^23^Na concentration from central necrosis to normal-appearing white matter suggests a correlation with tumor infiltration.

## Funding

S. R. is funded by the Physician-Scientist Programme of Heidelberg University, Faculty of Medicine.

## Disclosure statement

N.G.R.B. is currently employee of Siemens Healthcare in Erlangen, Germany.

## CRediT authorship contribution statement

**Sebastian Regnery:** Conceptualization, Validation, Formal analysis, Investigation, Visualization, Data curation, Writing - original draft, Writing - review & editing. **Nicolas G.R. Behl:** Methodology, Software, Validation, Visualization, Writing - original draft, Writing - review & editing. **Tanja Platt:** Methodology, Software, Validation, Writing - original draft, Writing - review & editing. **Nina Weinfurtner:** Investigation, Writing - original draft. **Paul Windisch:** Investigation, Writing - original draft. **Katerina Deike-Hofmann:** Investigation, Writing - original draft. **Felix Sahm:** Data curation, Writing - original draft. **Martin Bendszus:** Resources, Writing - original draft, Writing - review & editing. **Jürgen Debus:** Resources, Writing - original draft, Writing - review & editing. **Mark E. Ladd:** Methodology, Resources, Writing - original draft, Writing - review & editing. **Heinz-Peter Schlemmer:** Supervision, Resources, Writing - original draft, Writing - review & editing. **Stefan Rieken:** Writing - original draft, Writing - review & editing. **Sebastian Adeberg:** Conceptualization, Supervision, Writing - original draft. **Daniel Paech:** Conceptualization, Validation, Formal analysis, Investigation, Supervision, Project administration, Data curation, Writing - original draft, Writing - review & editing.

## Declaration of Competing Interest

The authors declare that they have no known competing financial interests or personal relationships that could have appeared to influence the work reported in this paper.
