# Supplementary material for: Ultra-high-field sodium MRI as biomarker for tumor extent, grade and IDH mutation status in glioma patients
Source: Neuroimage Clin. 2020 Sep 12;28:102427. doi: 10.1016/j.nicl.2020.102427 (PMC7527584; doi:10.1016/j.nicl.2020.102427)
Supplement: Supplementary data 1 [file mmc1.docx]

| **Parameter** | **T1-weighted  pre- and post- contrast** | **T2-weighted FLAIR** |
| --- | --- | --- |
| **TE [ms]** | 4.04 | 135 |
| **TR [ms]** | 1710 | 8500 |
| **FoV [mm²]** | 256x256 | 230x172 |
| **Matrix** | 512x512 | 256x192 |
| **Slice thickness [mm]** | 1 | 5 |

***Supplementary Table 1: 3T MRI protocols.*** *(FLAIR = fluid-attenuated inversion recovery, TE = echo time, TR = repetition time, FoV = field of view)*

| **Patient** | **^23^Na hotspot in edema** | **Corresponding FLAIR hotspot in edema** | **Edema ^23^Na concentration mean ± SD [mM]** |
| --- | --- | --- | --- |
| 1 | yes | no | 57 ± 11 |
| 2 | no | no | 75 ± 13 |
| 3 | no | no | 60 ± 17 |
| 4 | yes | partly | 62 ± 9 |
| 5 | no | no | 58 ± 11 |
| 6 | no | no | 60 ± 15 |
| 7 | no | no | 52 ± 17 |
| 8 | no | no | 67 ± 14 |
| 9 | no | no | 56 ± 10 |
| 10 | no | no | 68 ± 14 |
| 11 | yes | partly | 48 ± 11 |
| 12 | no | no | 33 ± 12 |
| 13 | yes | partly | 50 ± 12 |
| 14 | no | no | 60 ± 14 |
| 15 | no | no | 63 ± 12 |
| 16 | no | no | 49 ± 11 |
| 17 | yes | yes | 54 ± 8 |
| 18 | yes | yes | 64 ± 12 |
| 19 | no | no | 41 ± 10 |
| 20 | no | no | 45 ± 3 |
| 21 | no | no | 59 ± 11 |
| 22 | yes | no | 60 ± 11 |
| 23 | no | no | 63 ± 17 |
| 24 | no | no | 63 ± 14 |
| 25 | no | no | 57 ± 13 |
| 26 | no | no | 69 ± 17 |
| 27 | no | no | 39 ± 12 |
| 28 | no | no | 46 ± 12 |
| ∑ | yes: 7, no: 21 | yes: 2, partly: 3, no: 23 |  |

***Supplementary Table 2: ^23^Na concentration hotspots in tumor edema.*** *All ^23^Na images were semiquantitatively assessed for hotspot regions inside the tumor edema employing an image window that encompassed the patient’s individual mean signal inside edema ±2 standard deviations (SD). Regions inside the edema lying > 2 SD above the individual mean (window cut-off) and not abutting the border to contrast enhancing regions or considerably exceeding this border were defined as hotspot regions. The corresponding clinical T2-weighted fluid-attenuated inversion recovery (FLAIR) sequence was then inspected for correlating hotspots. Two patients presented unique hotspots on ^23^Na MRI (****red****), three patients showed increased hotspots on ^23^Na MRI compared to clinical FLAIR hotspots (****yellow****) and two patients showed approximately corresponding hotspot regions on both sequences (****blue****).*

***
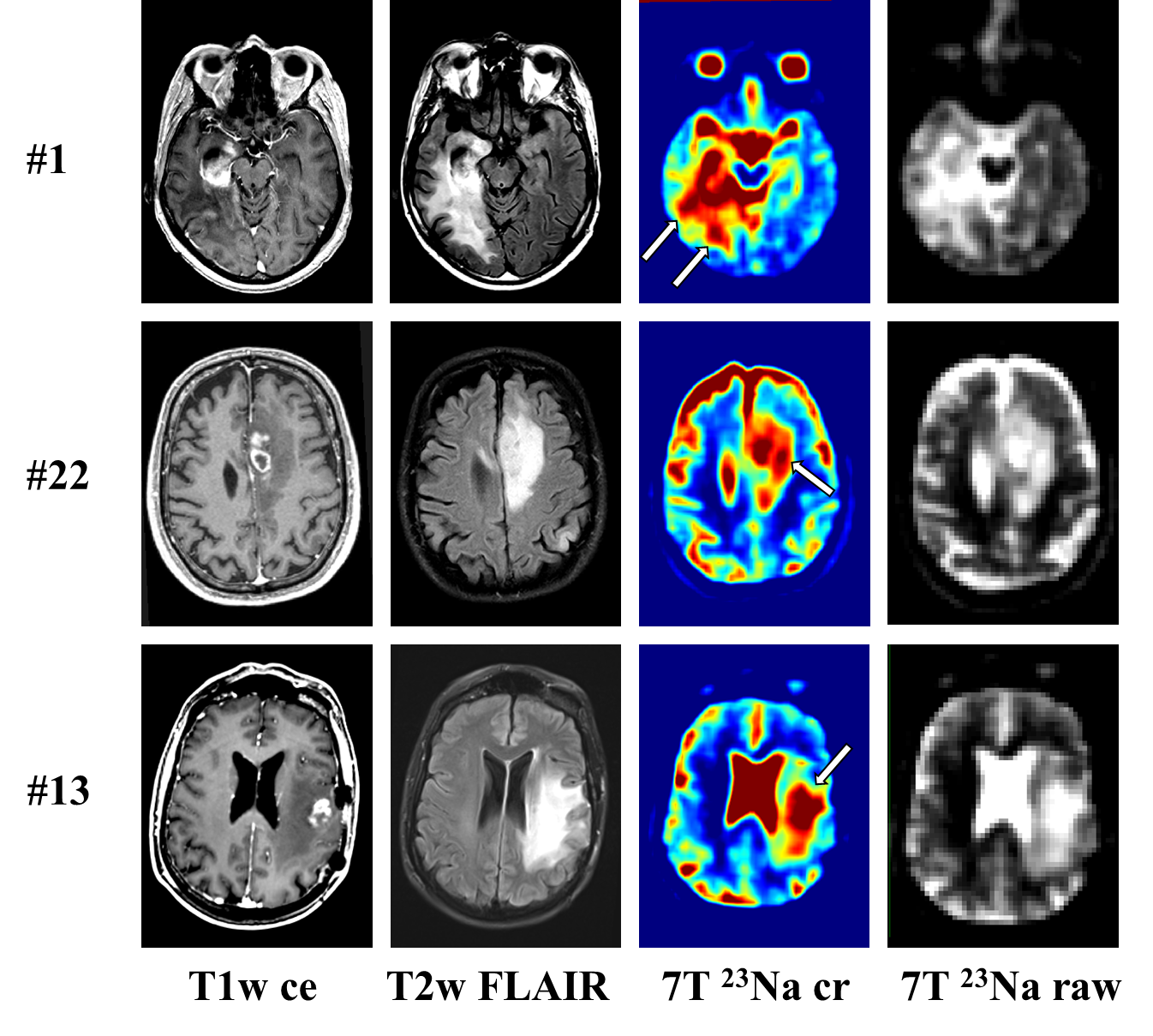
***

***Supplementary Figure 1: ^23^Na concentration hotspots in tumor edema.*** *Several exemplary 7T ^23^Na MR-images with corresponding clinical 3T imaging. The upper two rows show two patients with unique hotspot regions (white arrows) on ^23^Na imaging which are not adjacent to clinically contrast enhancing regions. The lower row shows one patient with a huge hotspot region inside tumor edema which partly correlates to the high edema signal on 3T FLAIR imaging, but partly also exceeds it (white arrow). ^23^Na images are shown in a window encompassing median edema ^23^Na concentration ± 2 standard deviations. (T1w ce = T1-weighted contrast enhanced, T2w FLAIR = T2-weighted fluid-attenuated inversion recovery, 7T ^23^Na cr = 7T ^23^Na imaging coregistered to clinical MRI including voxel interpolation, 7T ^23^Na raw = raw 7T ^23^Na imaging data).*


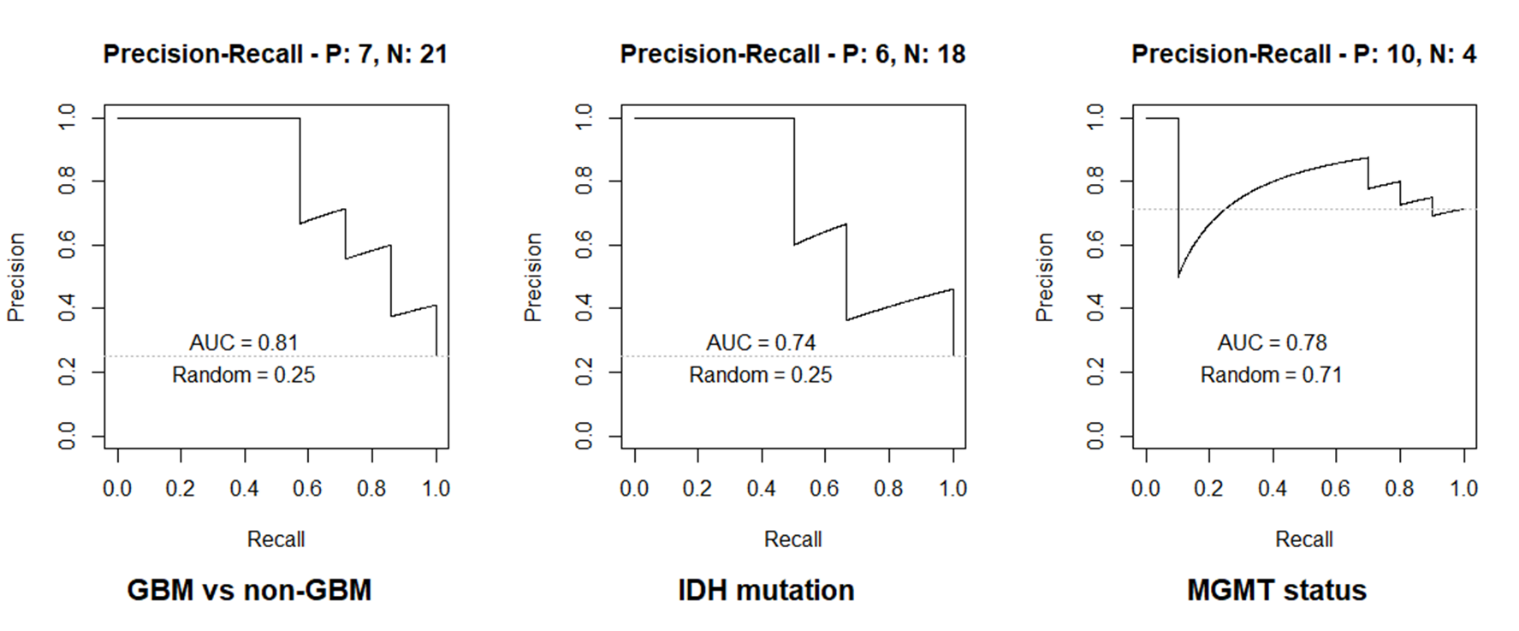


***Supplementary Figure 2: Precision Recall Curves.*** *Precision recall (PR) curves corresponding to the ROC curves for prediction of tumor grade (****left****), IDH mutation (****middle****) and MGMT status (****right****). When compared to ROC analysis, the predictive power, measured by precision and recall, is somewhat reduced due to group imbalance. Despite this, the total ^23^Na concentration still yields a fair-good prediction of tumor grade and IDH mutation, but only a poor prediction of MGMT status.*

*
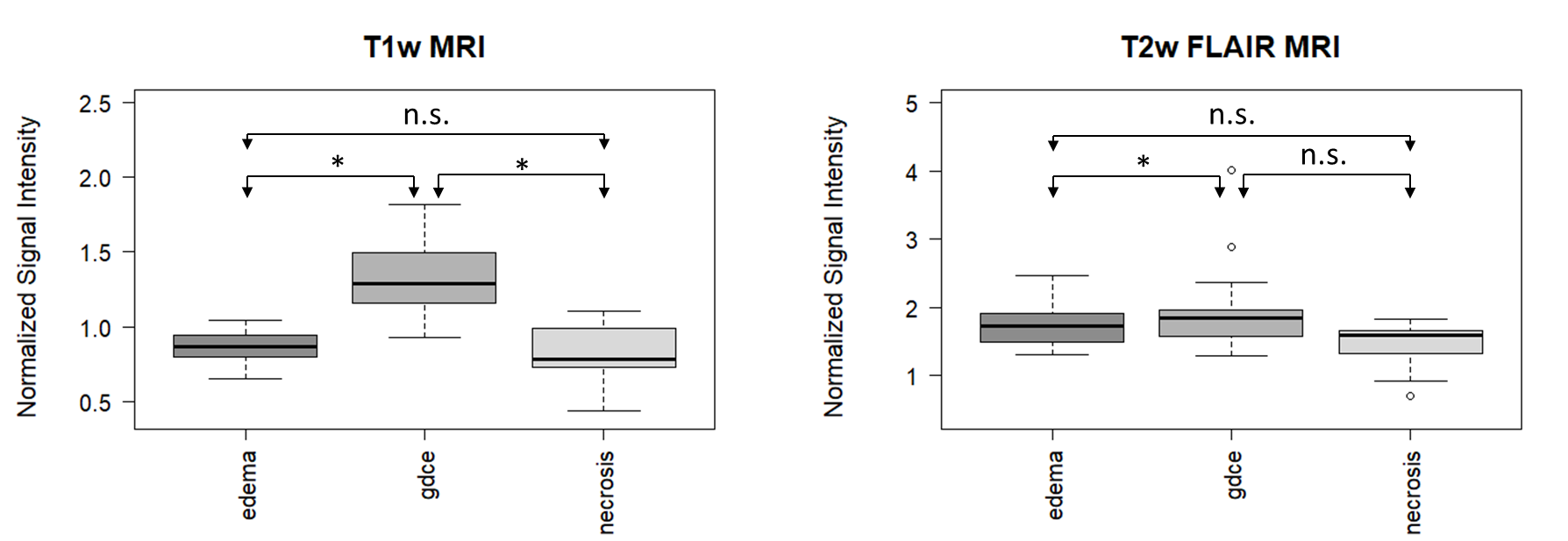
*

***Supplementary Figure 3: Analysis of signal intensities in different tumor subcompartments on clinical MRI.*** *All signal intensities were normalized to the normal appearing white matter signal.* *T1-weighted imaging showed significantly elevated signal intensity in* *Gadolinium-contrast enhancement (gdce) as compared to peritumoral edema and necrosis. No significant difference was found between the signal intensities of edema and necrosis (****left****). For T2-weighted fluid-attenuated inversion recovery (FLAIR) imaging, only gdce and edema differed significantly in their signal intensities (****right****). Neither clinical sequence shows a continuous signal change from central tumor parts towards the tumor periphery. (* = statistically significant with p < 0.05 but without correction for family-wise error rate).*

| **Subcompartments** | **T1w SI mean ± SD** | **p-value** | **T2w SI mean ± SD** | **p-value** |
| --- | --- | --- | --- | --- |
| **GDCE** | 1.34 [±0.25] | edema: 10^-7^ | 1.94 [± 0.6] | edema: 0.04 |
|  |  | necrosis: 10^-5^ |  | necrosis: 0.09 |
| **Edema** | 0.87 [± 0.09] | necrosis: 0.38 | 1.74 [± 0.28] | necrosis: 0.31 |
| **Necrosis** | 0.82 [± 0.21] | --- | 1.42 [± 0.39] | --- |

***Supplementary Table 3: Analysis of signal intensities in different tumor subcompartments on clinical MRI.*** *All signal intensities were normalized to the normal appearing white matter signal. P-values were calculated based on pairwise t-Tests. (T1w = T1-weighted MRI, T2w = T2-weighted FLAIR MRI, SI = signal intensity, SD = standard deviation, gdce = Gadolinium contrast enhancement).*


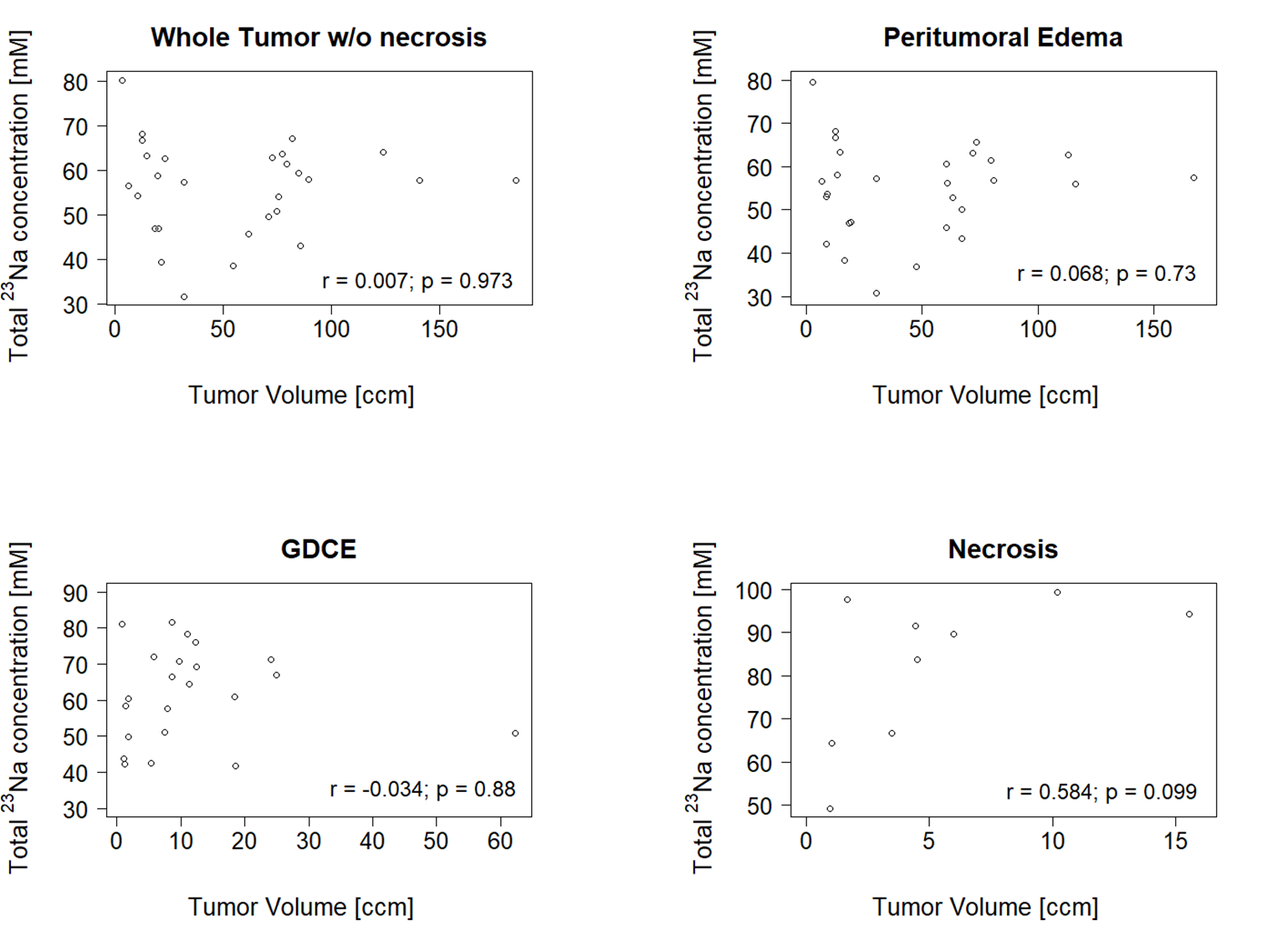


***Supplementary Figure 4: Correlation of segmented volumes with median total ^23^Na concentration.*** *No significant correlation was found between different tumor subregion volumes and median total ^23^Na concentration measured inside those regions: Whole tumor without necrotic areas (****top left****), peritumoral edema (****top right****), Gadolinium contrast enhancement (gdce) (****bottom left****), necrosis (****bottom right****).*


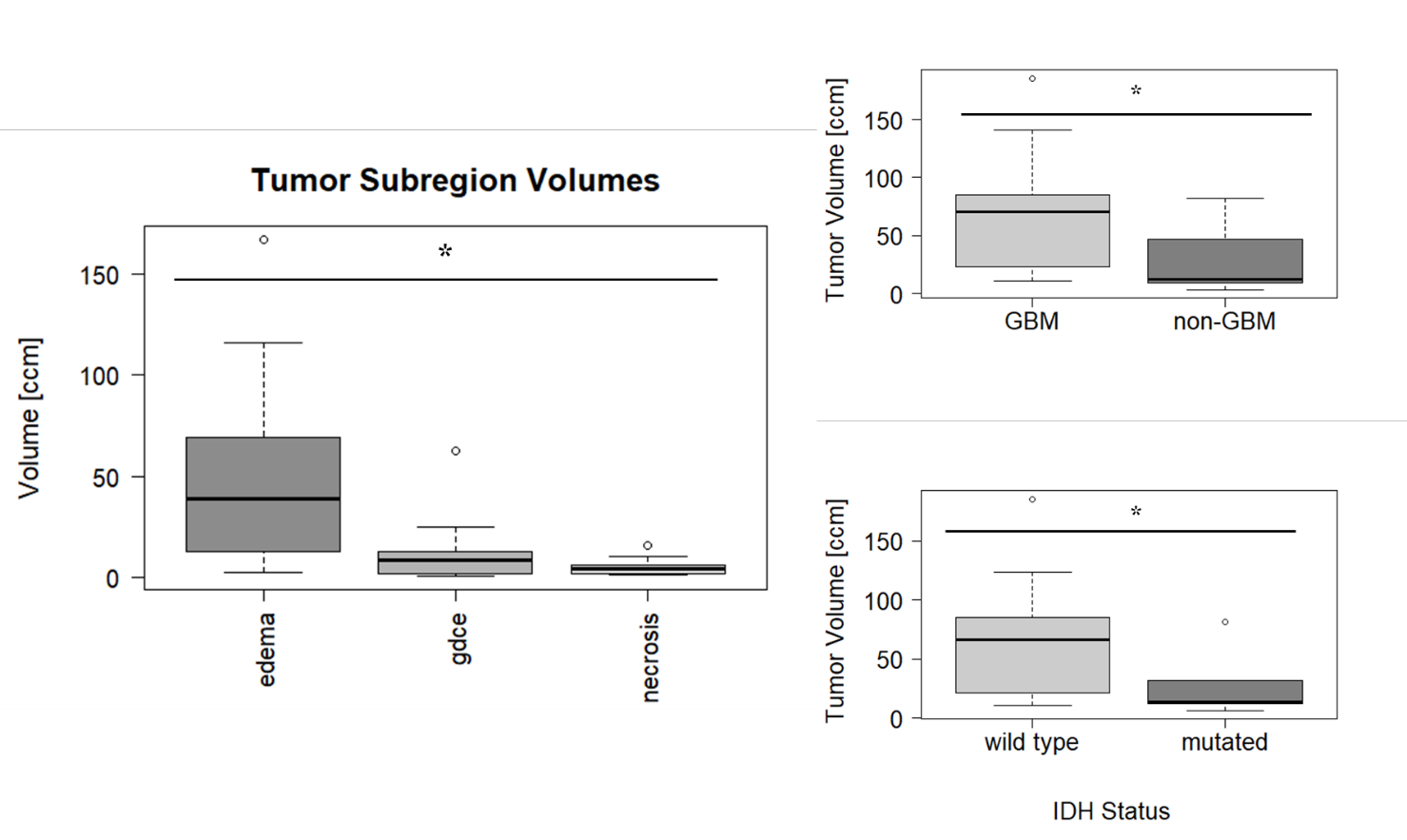


***Supplementary Figure 5: Volumetric analysis of tumor subcompartments and different histopathological groups.*** *The peritumoral edema showed the highest volumes (47.52 ± 40.48 ccm), followed by* *Gadolinium-contrast enhancement (gdce, 11.7 ± 13.39 ccm) and necrosis (*5.3 ± 4.79 *ccm). The differences in segmentation volumes reached statistical significance for all comparisons (****left****). Non-GBM tumors (*30.19 ± 34.76 ccm) *and IDH mutated tumors (*26.69 ± 28.35 ccm) *showed significantly lower tumor volumes compared to their GBM (*65.55 ± 45.12 ccm) *and IDH wild type (*62.83 ± 44.53 ccm) *counterparts, respectively (****right****). (* = statistically significant with p < 0.05 and without correction for family-wise error rate).*
